# Intestinal probiotics *E. coli* Nissle 1917 as a targeted vehicle for delivery of p53 and Tum-5 to solid tumors for cancer therapy

**DOI:** 10.1186/s13036-019-0189-9

**Published:** 2019-06-28

**Authors:** Lian He, Huijun Yang, Jianli Tang, Zhudong Liu, Yiyan Chen, Binghua Lu, Haocheng He, Sijia Tang, Yunjun Sun, Fei Liu, Xuezhi Ding, Youming Zhang, Shengbiao Hu, Liqiu Xia

**Affiliations:** 1 Hunan Provincial Key Laboratory of Microbial Molecular Biology, State Key Laboratory of Developmental Biology of Freshwater Fish, College of Life Science, Hunan Normal University, Changsha, 410081 People’s Republic of China; 2 School of Basic Medical Science, Changsha Medical University, Changsha, 410298 People’s Republic of China

**Keywords:** *E.coli* Nissle 1917, Targeted cancer therapy, p53, Tum-5, Gene therapy

## Abstract

Traditional cancer therapies, such as surgery treatment, radiotherapy, and chemotherapy, often fail to completely eliminate tumor cells in an anaerobic microenvironment of tumor regions.

In contrast to these traditional cancer therapies, the use of targeted delivery vectors to deliver anticancer genes or antitumor drugs to hypoxic areas in tumors is the most clinically promising cancer treatment with rapid development in recent years. In this study, *E.coli* Nissle 1917 (EcN), an intestinal probiotic, was utilized as a targeted transport vector to deliver p53 and Tum-5 protein to tumor hypoxic regions. The tumor-targeting characteristics of EcN were investigated using luciferase *Lux*CDABE operon, and the results demonstrated that EcN could specifically accumulate in the solid tumor areas of SMMC-7721 tumor-bearing BALB/c nude mice. The Tum 5-p53 bifunctional proteins were initially constructed and then delivered to solid tumor regions by using the targeted transporter EcN for cancer therapy. The antitumor effect and safety of three engineered bacteria, namely, EcN (Tum-5), EcN (p53), and EcN (Tum 5-p53), were also examined. The calculated tumor volume and tumor weight indicated that these three engineered bacteria could inhibit the growth of human hepatoma SMMC-7721 cells, and the antitumor effect of EcN (Tum 5-p53) expressing the Tum 5-p53 fusion protein was significantly better than those of EcN (Tum-5) and EcN (p53) alone. Immunofluorescence demonstrated that the expression of Ki-67, a nuclear proliferation-related protein, was inhibited in the tumor areas of the groups treated with the engineered bacteria, whereas the expression of caspase-3 was upregulated. The expression trends of Ki-67 and caspase-3 were consistent with the different antitumor efficacies of these three engineered bacteria. EcN did not elicit obvious side effects on mice. This research not only provids a foundation for tumor-targeted therapy but also contributes greatly to the development of antitumor agents and anticancer proteins.

**Electronic supplementary material:**

The online version of this article (10.1186/s13036-019-0189-9) contains supplementary material, which is available to authorized users.

## Introduction

Cancer poses a serious threat to human life and health [1–3]. At present, metastatic tumors are the main cause of death in many cancer patients after treatment. Traditional cancer treatment methods, such as radiotherapy and chemotherapy, often result in low survival rates or severe side effects on normal cells, which limits the therapeutic effect due to the development of drug resistance and lack of tumor specific drugs [4]. Currently, gene therapy is a promising cancer treatment method for treating all types of cancers, which mainly promotes the development of antitumor effects by delivering therapeutic proteins or medicines to patients [5, 6].

As a delivery vehicle for gene therapy, bacteria can effectively deliver DNA to cells or targeted tissues [7]. The main advantage of using bacteria for cancer treatment is that certain bacteria have excellent tumor-targeting properties on tumor tissues. The mechanism of bacterial accumulation in the tumor areas depends on its tolerance to oxygen. Obligate anaerobes (e.g., *Clostridium* [8] and *Bifidobacterium* [9]) cannot survive under aerobic conditions. During tumor treatment, bacterial spores only germinate in the tumor regions of the hypoxic microenvironment [10]. The anaerobic environment in the tumor tissues is very special, resulting in the rapid accumulation of obligate anaerobes in the tumor necrosis areas [11]. Malmgren et al. injected *Clostridium tetani* into tumor-bearing mice. They found that the bacteria could colonize the hypoxic regions of tumor necrosis and that the survival time of mice in the treatment group was significantly prolonged [12]. The tumor-targeting mechanism of facultative anaerobes, such as *E.coli* Nissle 1917 [13–15] and *Salmonella* [16, 17], is very complex. Facultative anaerobes can accumulate in the tumor areas and it may be caused by five interacting mechanisms: chaotic vasculature system in the tumor areas captures bacteria [18]; inflammatory response occurs when bacteria enter the tumor area [19]; chemokines secreted from tumor regions have chemotactic effects on bacteria [20, 21]; bacteria can preferentially grow in the tumor microenvironment [22]; and bacteria are not easily cleared by the body’s immune system in the tumor immunosuppressive environment [23].

p53 is a tumor suppressor protein that can control responses to a variety of cellular stresses, including DNA damage, hypoxia, and oncogene activation [24, 25]. It acts as a transcription factor and binds to specific DNA sequences [26, 27]. Previous studies indicated that the function of p53 is not only involved in apoptosis, senescence and cell cycle arrest, but it also plays an important role in metabolism, necrosis, autophagy, active oxygen accumulation, and stem cell maintenance [28]. Moreover, our previous studies have successfully demonstrated that the anti-angiogenic protein Tum-5 can exert antitumor effect by inhibiting neovascularization in the tumor areas [13]. Given that p53 and Tum-5 proteins can inhibit tumor growth through different mechanisms, we envisaged that the combination of the ability of p53 protein to directly induce apoptosis in tumor cells and the anti-angiogenic function of Tum-5 could be a potential gene therapy for cancer treatment.

**Fig. 1:**
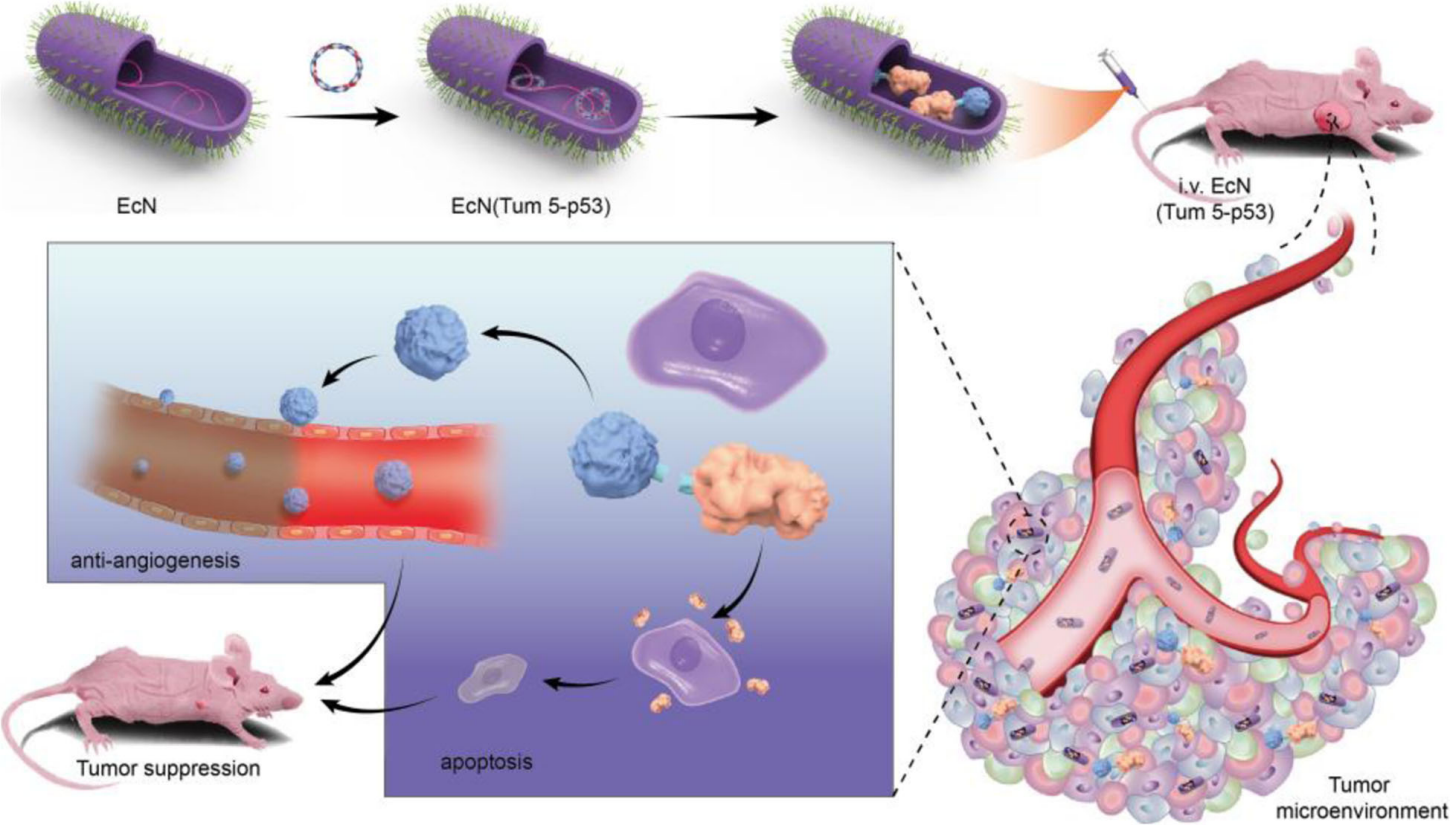
EcN (Tum 5-p53) probiotics for cancer therapy**.** The engineered bacteria (purple) can specially accumulate in tumor areas of BALB/c mice bearing SMMC-7721 tumor after intravenous injection. The recombinant protein could be cleaved by matrix metalloproteinases that were highly expressed in the tumor regions, thereby inhibiting the growth of tumor cells through different anti-tumor mechanisms

Therefore, p53 and Tum-5 fusion protein was constructed using matrix metalloproteinase (MMP) cleavage site (PLGLWA) [29–31] as a fusion gene linker, thereby enabling tumor-targeting engineered bacteria to produce bifunctional proteins capable of directly inducing apoptosis and inhibiting angiogenesis. The intestinal probiotic EcN was used as a gene vehicle to deliver the anticancer protein p53 and the anti-angiogenic factor Tum-5 to the tumor hypoxic regions for cancer treatment (Fig. 1). The results demonstrated that the engineered bacteria succeeded in inhibiting the growth of human hepatoma SMMC-7721 cells in tumor-bearing BALB/c nude mice, which resulted in the significant inhibitory effects of engineered EcN on the growth of orthotopic hepatoma cancers without any notable toxicity.

## Results

### Expression and biological activity analysis of p53 protein in vitro

**Fig. 2:**
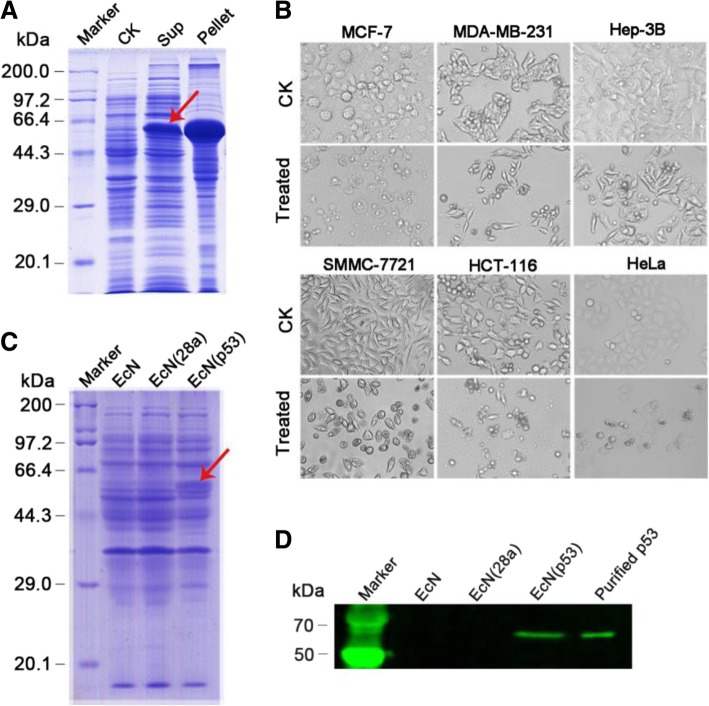
Efficient expression of p53 protein in *E. coli* BL21 (DE3) and probiotic EcN**. a** p53 expression levels in cell lysates (Pellet) or supernatants (Sup) were detected by subjecting *E. coli* BL21 (DE3) bearing *p53*-gene-expressing plasmid or empty plasmid (CK) to SDS-PAGE after IPTG induction and ultrasonication. **b** p53 protein could inhibit the proliferation of various types of cancer cells. PBS (CK) and 40 μg/mL recombinant p53 protein were coincubated with MCF-7, MDA-MB-231, Hep-3B, SMMC-7721, HCT-116, HeLa tumor cells, and CCK-8 solution was used to calculate the inhibitory effect of the recombinant protein on tumor cells. SDS-PAGE (**c**) and Western blot analysis (**d**) of p53 expression in EcN, EcN (28a), and EcN (p53)

The anticancer gene *p53* was amplified from the mRNA of MCF-7 cells and cloned in the expression vector pET28a (Additional file 1: Figure S1–S3). The p18 peptide of azurin was placed on the N-terminus of p53 protein, and the p18-p53 fusion protein was successfully solubly expressed in *E.coli* BL21 (DE3) (Fig. 2a). Western blot analysis and mass spectrum were performed to determine the correct expression of p53 (Additional file 1: Figure S4). The tumor cells were treated with 40 μg/mL p53 protein for 48 h, and the inhibitory rates of the recombinant protein on MCF-7, MDA-MB-231, and Hep-3B cells reached 52.16, 38.03, and 31.89%, respectively (Fig. 2b and Additional file 1: Figure S4). The inhibition rates of SMMC-7721, HCT-116 and HeLa cells reached 61.84, 69.70 and 77.98%, respectively (Fig. 2b). To enable p53 protein to reach the solid tumor area and successfully exert its antitumor activity, it is necessary to use the targeted transport function of the tumor-targeting bacteria EcN. Based on the vector pET28a-Pvhb-pelB-SUMO-Tum 5 from previous studies, p53 was placed under the hypoxia promoter Pvhb using Red/ET homologous recombination technology (Additional file 1: Figures S7 and S8). SDS-PAGE and Western blot indicated that p53 protein was efficiently expressed in EcN (Fig. 2c and d).

### Construction of the engineered EcN for targeted delivery of tum 5-p53 protein to hypoxic regions of solid tumors

**Fig. 3:**
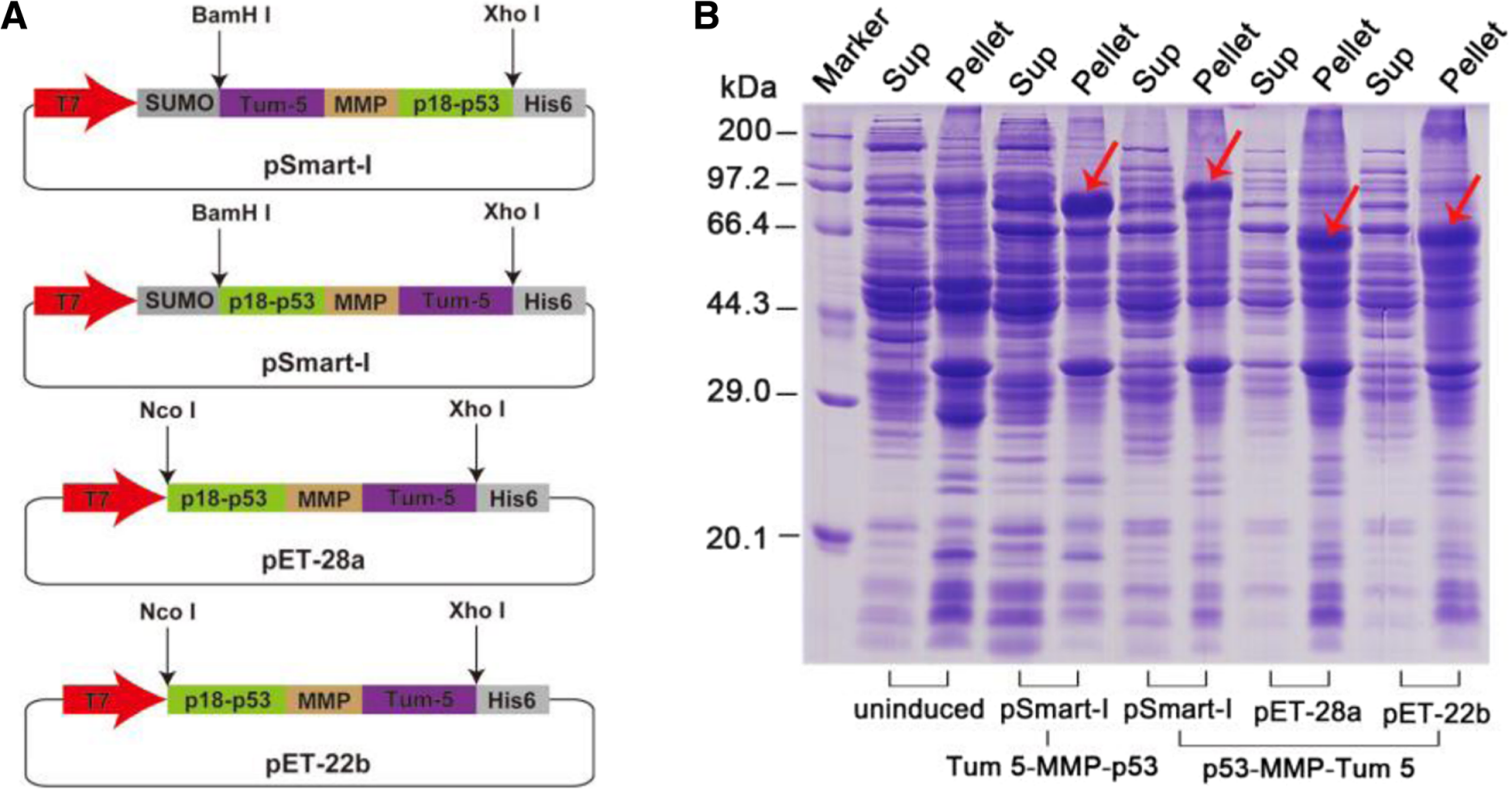
Construction and expression of Tum 5-p53 bifunctional proteins**. a** Construction map of p53 and Tum-5 fusion expression vectors. T7: T7 promotor; SUMO: small ubiquitin-related modifier; MMP: matrix metalloproteinase (MMP) cleavage site (PLGLWA); p18: the amino acid 50–67 of azurin. **b** Coomassie Brilliant Blue staining of SDS-PAGE showing fusion protein expression in *E. coli* BL21 (DE3). Recombinant proteins expression in cell lysates (Pellet) or supernatants (Sup) was detected by SDS-PAGE of *E. coli* BL21 (DE3) bearing recombinant plasmid after IPTG induction and ultrasonication

To construct bifunctional proteins that can directly induce apoptosis and inhibit angiogenesis, the angiogenesis inhibitor Tum-5 and the anti-oncoprotein p53 were fused with an MMP cleavage site (PLGLWA) as the linker (Fig. 3a, Additional file 1: Figure S8, Additional file 1: Figure S9). Protein soluble analysis suggested that both p53-MMP-Tum 5 and Tum 5-MMP-p53 fusion proteins were successfully expressed in *E.coli* BL21(DE3) host strains (red arrow), but the locations of p53 and Tum-5 at the N-terminus or C-terminus and the different tags on the vector significantly influenced solubility of the fusion protein (Fig. 3b). When the Tum-5 protein was located at the C-terminus of the fusion protein, the p53-Tum 5 fusion protein could only be expressed as an inclusion body on the pET28a, pET22b, and pSmartI vectors. However, when Tum-5 was located on the fusion protein at the N-terminus, the Tum 5-p53 fusion protein was expressed in a partially soluble form on the pSmartI vector (Fig. 3b). The recombinant Tum 5-p53 fusion protein exhibited a good inhibitory effect on human hepatoma SMMC-7721 cells and human cervical cancer HeLa cells in vitro *i*n a significantly dose-dependent manner (Additional file 1: Figure S10).

**Fig. 4:**
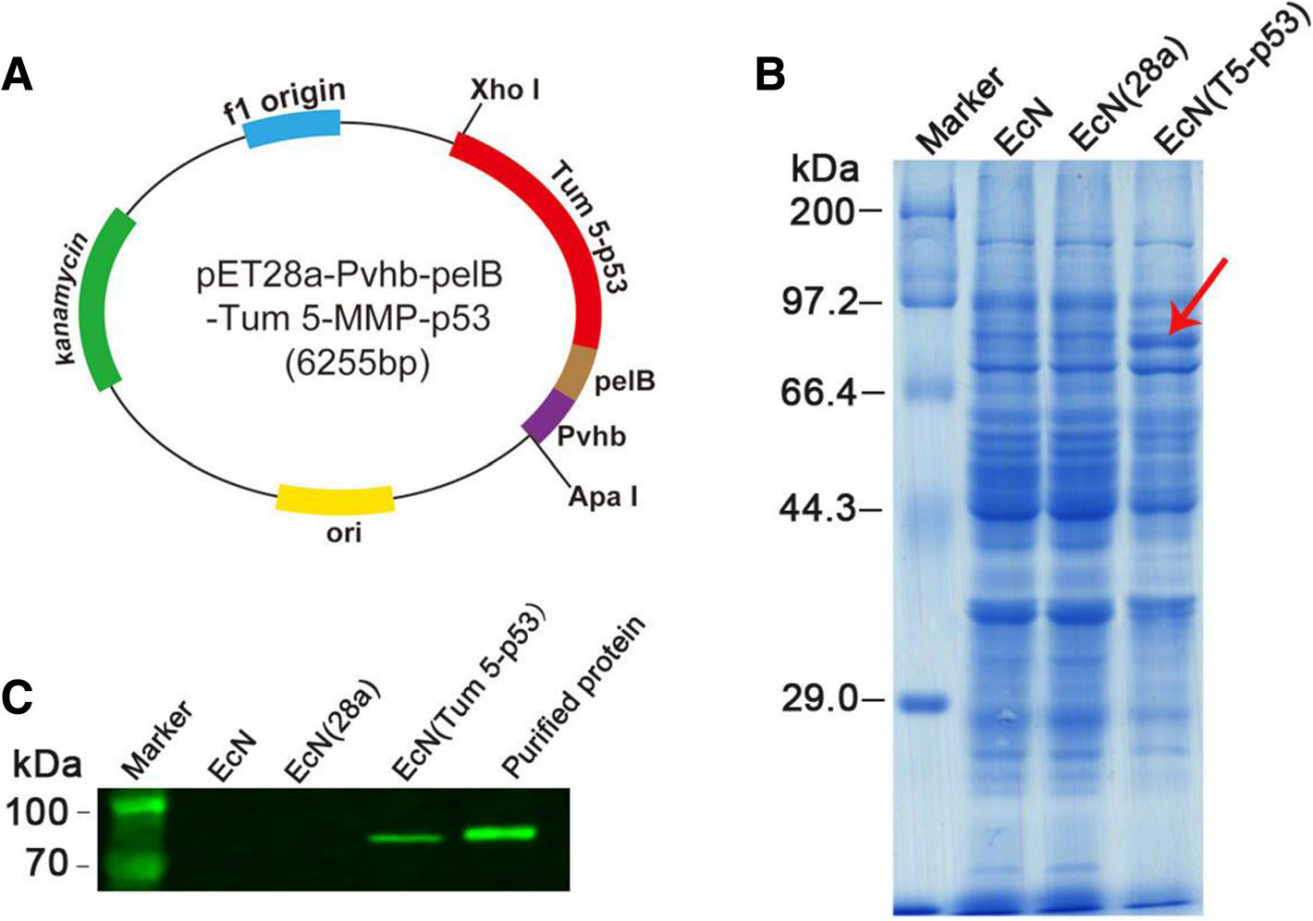
Tum 5-p53 bifunctional protein was successfully expressed in tumor-targeting bacteria EcN. **a** Map of recombination plasmid containing Tum 5-p53 gene. Pvhb: promoter of the *Vitreoscilla* hemoglobin gene; pelB: the signal peptide from the pelB gene of *Erwinia carotovora*. SDS-PAGE (**b**) and Western blot analysis (**c**) of Tum 5-p53 bifunctional protein expression in EcN, EcN (28a), and EcN (Tum 5-p53)

In this study, the pET28-Pvhb-pelB-SUMO-Tum 5-MMP-p53 vector was constructed using overlapping PCR (Additional file 1: Figure S11). The fragment was ligated into the pET28a vector to obtain the hypoxia expression vector (Fig. 4a and Additional file 1: Figure S12). Sequencing analysis indicated that the low-oxygen expression vector was successfully constructed. The low-oxygen expression vector was electrotransformed into EcN, and an antitumor engineered strain EcN (Tum 5-p53) was successfully obtained. The results of SDS-PAGE suggested that the EcN (Tum 5-p53) exhibited obvious targeted band (red arrow) at around 80 kDa (Fig. 4b). The band was consistent with the expected molecular weight, indicating that the Tum 5-p53 protein was successfully expressed in EcN. Western blot analysis demonstrated that the bifunctional protein was efficiently expressed in EcN (Fig. 4c).

### EcN is specifically accumulated in the tumor microenvironment of human hepatocellular carcinoma SMMC-7721 BALB/c nude mice

**Fig. 5:**
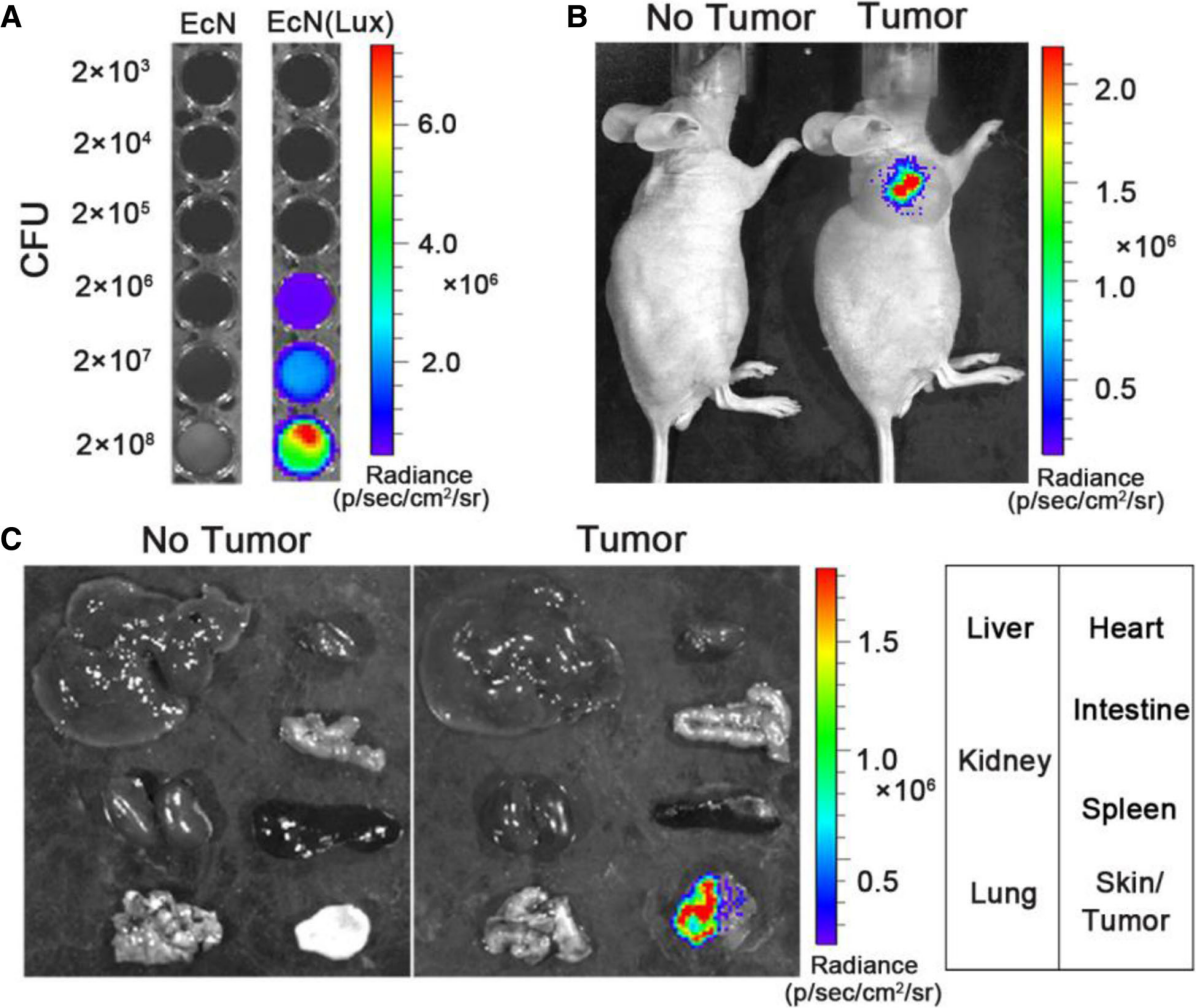
Targeting of EcN to tumor tissues after systemic administration**. a** Fluorescent luminescence detection of EcN (Lux) at different concentrations. **b** EcN (Lux) was systematically injected to BALB/c nude mice bearing SMMC-7721 tumor cells. For control, EcN (Lux) was injected to healthy BALB/c nude mice with no tumor. Whole body distributions of the injected EcN (Lux) were observed using in vivo imaging system spectrum 72 h after the injection. **c** Spleen, liver, kidney, lung, heart, intestine, and tumor tissues were isolated to investigate the bioluminescence signals in different organs

For non-invasive live detection, a reporter plasmid conferring constitutive *Lux* expression was transformed into the EcN (Fig. 5a). When the tumor volume grows to an appropriate size, the normal nude mice and the tumor bearing nude mice were intravenously injected with 5 × 10^6^ CFUs/100 μL of EcN (Lux) to assess the in vivo distribution of strain in mice. An in vivo imaging system was used to observe the colonization of bacteria in human hepatocellular carcinoma SMMC-7721 tumor-bearing BALB/c nude mice. The results indicated that a clear bioluminescence signal was detected in the tumor area 72 h after bacterial injection of tumor-bearing mice, whereas no signal was detected in normal nude mice (Fig. 5b). After the mice were sacrificed, they were dissected to obtain tumors, liver, kidney, spleen, lung, heart, small intestine, and skin. After intravenous injecting EcN (Lux) into normal nude mice for 72 h, no signal was detected in all organs, which indicated that the bacteria were basically cleared by the body’s immune system. However, a strong bioluminescence signal appeared in the tumor tissue of tumor-bearing nude mice, but not in other organs (Fig. 5c). The above results indicated that EcN has excellent targeting ability to the solid tumor regions of human hepatocellular carcinoma SMMC-7721 BALB/c nude mice. The EcN bacteria can rapidly accumulate in the tumor microenvironment but not in other organs after being injected into nude mice.

### EcN (tum 5-p53) significantly inhibited SMMC-7721 proliferation in BALB/c nude mice

**Fig. 6:**
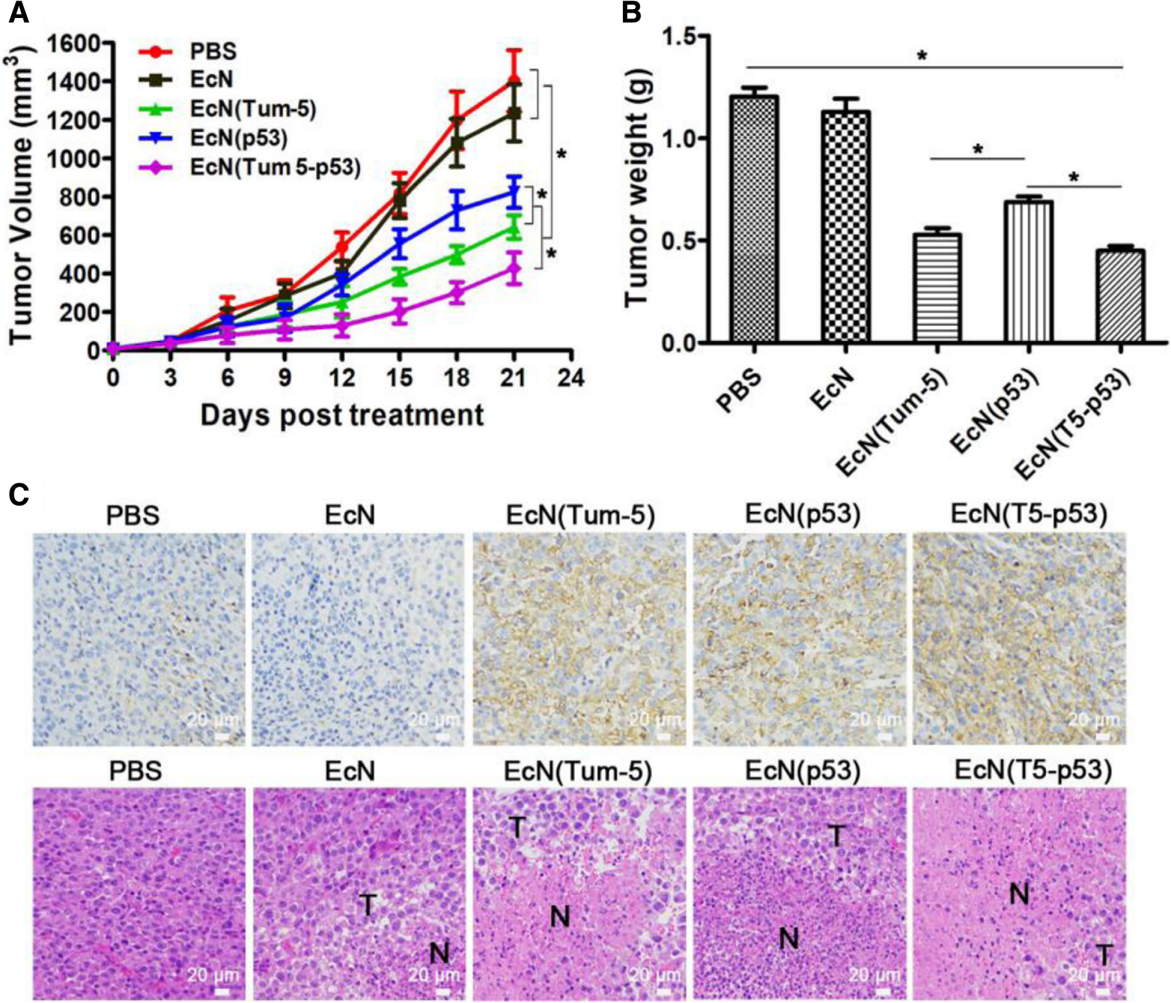
Tumor therapeutic effects and intra-tumor distribution of therapeutic proteins**. a** BALB/c nude mice bearing SMMC-7721 tumors were intravenously injected with PBS, EcN, EcN (p53), EcN (Tum-5), or EcN (Tum 5-p53) weekly. The tumor volumes of the mice in each group (*n* = 6) were measured from the beginning of treatment. **b** At the end of the treatment, the mice were sacrificed and tumor tissues were excised and weighed. SMMC-7721 tumors were significantly inhibited by EcN (Tum-5), EcN (p53), EcN (Tum 5-p53) compared with the PBS or EcN group. **c** Identification of the distribution of Tum 5-p53 protein in tumor tissues by immunohistochemistry and H&E staining of tumor tissues (200×)

The tumor-bearing mice were randomly divided into five groups, and each group was intravenously injected with EcN, EcN (Tum-5), EcN (p53), and EcN (Tum 5-p53) at a dose of 5 × 10^6^ CFUs/100 μL or sterile PBS for animal experiment. The longest and widest diameters of tumors in each group of tumor-bearing nude mice were measured. The tumor volume was calculated according to the formula, and the time–tumor curve reflecting the tumor growth trend was plotted. The tumor volumes of the EcN (Tum-5), EcN (p53), and EcN (Tum 5-p53) groups were significantly reduced (*P* < 0.05) compared with those of the PBS group and the EcN group (Fig. 6a). No significant difference was observed in the tumor volume between the EcN group and the PBS group. In the three treatment groups, the antitumor engineered strain EcN (Tum 5-p53) expressing the Tum 5-p53 fusion protein was significantly superior to the EcN (Tum-5) expressing the Tum-5 protein alone and EcN (p53) expressing the p53 protein alone (*P* < 0.05), whereas the therapeutic effect of EcN (Tum-5) on human hepatocellular carcinoma SMMC-7721 was slightly higher than that of EcN (p53) (*P* < 0.05) (Fig. 5a and b). Compared with PBS and EcN groups, the tumor volume and tumor weight of the EcN (Tum-5)-, EcN (p53)-, and EcN (Tum 5-p53)-treated groups of mice were obviously improved. According to the tumor volume, the tumor inhibition rates of the EcN (Tum-5), EcN (p53), and EcN (Tum 5-p53) groups were 54.28, 41.22, and 69.47%, respectively (Fig. 6a and b). According to the weight of the tumor, the tumor inhibition rates of the EcN (Tum-5), EcN (p53), and EcN (Tum 5-p53) groups were 55.83, 47.92, and 62.5%, respectively (Fig. 5a and b). These results indicated that EcN (Tum-5), EcN (p53), and EcN (Tum 5-p53) could significantly inhibit the growth of human hepatocellular carcinoma SMMC-7721 tumors (*P* < 0.05).

At the end of the experiment, the mice were sacrificed and the tumor tissues were obtained. Immunohistochemistry was performed using Anti-6 × His polyclonal antibody. Significant brown–yellow signals were detected in the tumor tissue of the EcN (Tum-5), EcN (p53), and EcN (Tum 5-p53) groups, whereas no positive signal was detected in the tumor sections of the PBS and EcN groups (Fig. 6c), indicating that the recombinant protein was continuously and efficiently expressed in the tumor area. The results of H&E staining showed that necrosis was observed in tumor tissues of mice treated with EcN, EcN (Tum-5), EcN (p53), and EcN (Tum 5-p53), and inflammatory cells infiltrated into the tumor area. The tumor tissue morphology of the PBS group was complete (Fig. 6c).

### Study on the antitumor mechanism of the three engineered strains

**Fig. 7:**
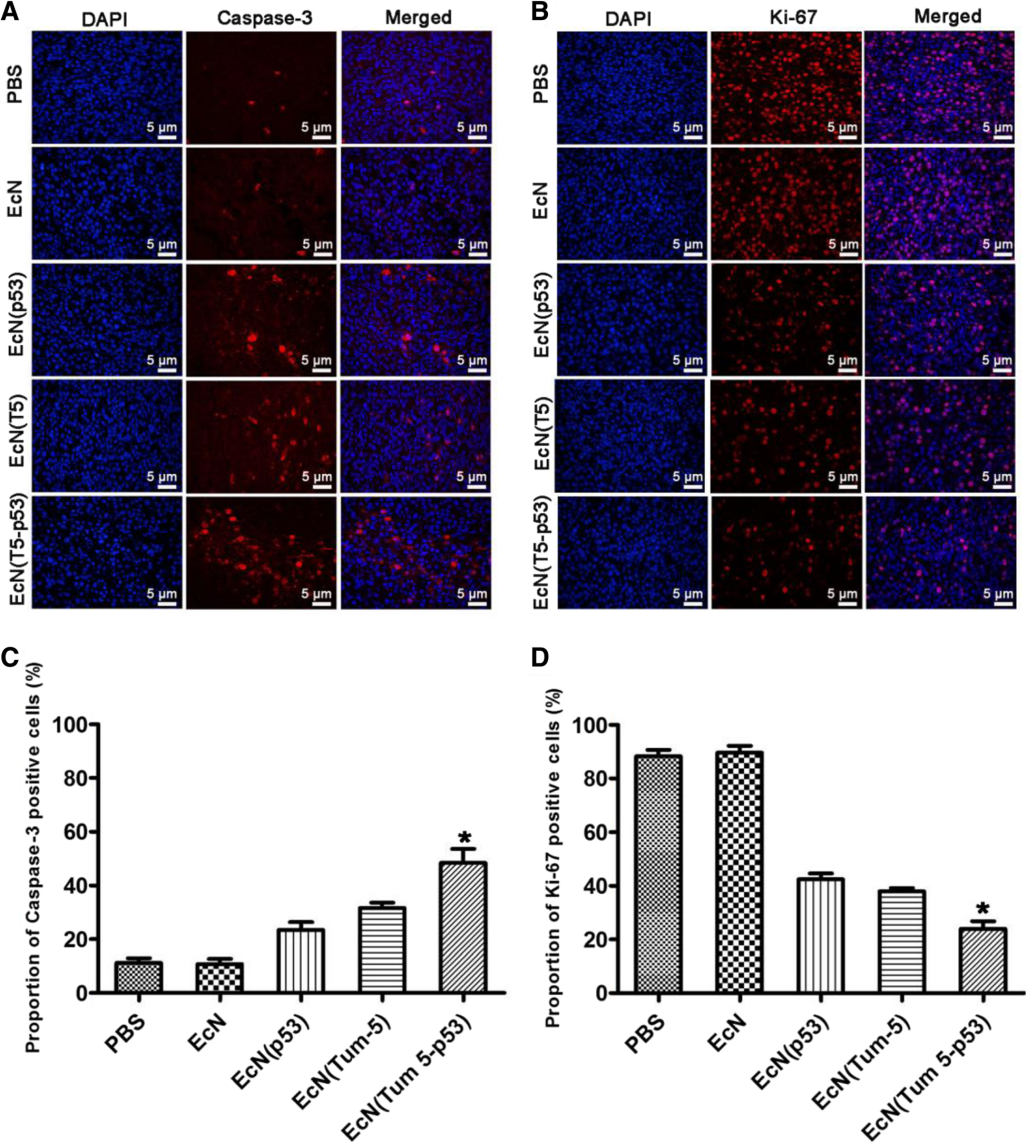
Immunofluorescence assay of caspase and Ki-67 expression in the tumor areas (400×). Images of tumor tissues isolated from PBS-, EcN-, EcN (p53)-, EcN (Tum-5)-, or EcN (Tum 5-p53)-treated BALB/c nude mice bearing SMMC-7721 tumors, which were stained for caspase-3 (**a**) and Ki-67 (**b**) after intravenous injection, and the cell nucleus is stained in blue (Hoechst). Quantitative analysis of caspase-3 (**c**) and Ki67 (**d**) positive cells in the tumor areas of mice in each group (*P<0.05)

Caspase family proteins are key signaling molecules in the process of apoptosis. Among them, caspase-3 is a key apoptosis executor [32]. It is activated by the death signal and catalyzes the specific cleavage of a variety of apoptosis-related proteins in cells. It plays an important role in multiple pathways of apoptotic signal transduction. Numerous studies have shown that caspase-3, a marker of apoptosis, can be used to assess the degree of apoptosis in a tissue or cell [33]. Caspase-3 was labeled with red using a specific antibody. Immunofluorescence did not detect red signals in the tumor areas of the PBS and EcN groups. In the EcN (p53)- and EcN (Tum-5)-treated groups, a small amount of red signal were observed in some tumor regions. However, a large amount of caspase-3 red signals were found in the tumors of the EcN (Tum 5-p53) group (Fig. 7a and c), indicating that gene combination therapy can induce tumor tissues to undergo apoptosis.

Ki-67 protein (also known as MKI67) is a nuclear antigen closely related to mitosis and associated with proliferating cells [34]. It is present in all phases of cell cycle activity (G1, S, G2, and mitosis). However, it does not exist in resting cells (G0). Ki-67 is a biological indicator for detecting a variety of malignancies and can be used to detect the cell proliferation activity of malignant tumors [35]. The results of immunofluorescence indicated that Ki-67 expression existed in nearly all tumor cells in the PBS and EcN-treated groups (the blue signal basically coincided with the red signal). However, in the EcN (p53), EcN (Tum-5), and EcN (Tum 5-p53)-treated groups, the expression of Ki-67 in the tumor region decreased, and only a few cells in the tumor region of EcN (Tum 5-p53) group showed red Ki-67 (Fig. 7b and d). The results indicated that the gene combination therapy can significantly inhibit the proliferation activity of tumor cells.

### EcN probiotics exhibit a safety profile upon systemic infection

**Fig. 8:**
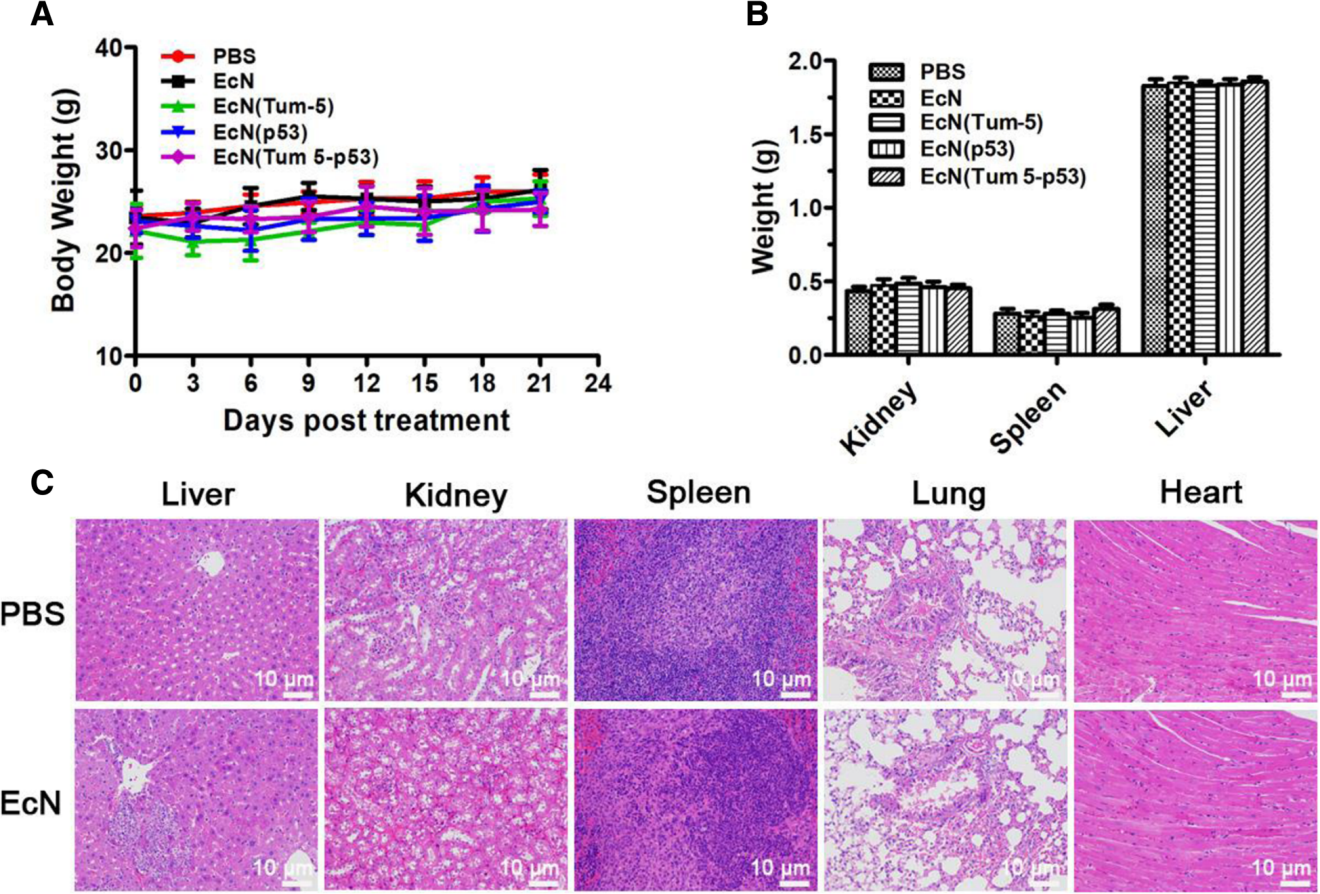
In vivo toxicity assay**.** BALB/c nude mice were weekly treated with PBS, EcN, EcN (p53), EcN (Tum-5), and EcN (Tum 5-p53). The weights of the mice were recorded every 2 days (**a**). At the end of the treatment, the livers, spleens, and kidneys of five mice were excised and weighed (**b**). Histology of liver, spleen, kidney, lung, and heart extracted from the mice (**c**)

During the treatment, tumor-bearing nude mice of the PBS, EcN, EcN (Tum-5), EcN (p53), and EcN (Tum 5-p53) groups were weighed every 3 days to assess whether the bacteria had toxic side effects on the nude mice. The results showed no significant changes in the body weight of nude mice in the PBS group during the three intravenous injections, indicating that the intravenous administration did not cause significant discomfort in the nude mice (Fig. 8a). The body weight changes of nude mice in the EcN-, EcN (Tum-5)-, EcN (p53)-, and EcN (Tum 5-p53)-treated groups were similar to those in the PBS group, which showed a slowly increasing trend (Fig. 8a). The liver, kidney, and spleen of the animals were weighed. Statistical analysis showed that there was no significant weight change in the liver, kidney, and spleen in each group (Fig. 8b). After the end of the treatments, the mice were sacrificed, and the liver, kidney, spleen, lung, and heart of the mice were dissected. H&E staining showed no significant differences in the pathological morphology between the PBS and EcN groups (Fig. 8c), and 5 × 10^6^ CFUs of EcN is basically safe for nude mice. These results indicated that intravenous injection of EcN has no obvious toxic side effects in tumor-bearing nude mice.

## Discussion

Using targeted transport of bacteria to target the delivery of antitumor proteins or anticancer drugs to the tumor area is a promising cancer treatment method [36, 37]. In this study, EcN was used to deliver anticancer protein p53 and anti-angiogenic factor Tum-5 to the solid tumor areas. The engineered bacteria had a significant inhibitory effect on the tumor growth of tumor-bearing mice and achieved excellent therapeutic effect on tumors. However, some unsolved problems remain to be investigated further.

Recombinant bifunctional Tum 5-p53 protein that can induce apoptosis and inhibit angiogenesis has significant inhibitory effects on the growth of human hepatoma SMMC-7721 cells and human cervical cancer HeLa cells. The tether PLGLWA of the Tum 5-p53 fusion protein has been shown to be an MMP cleavage site and can be successfully cleaved by MMPs [29–31]. We envisage that the Tum 5-p53 fusion protein is targeted to the tumor region, and the fusion protein is cleaved by the secreted MMPs in the tumor region. On the one hand, the p53 and Tum-5 proteins can exert antitumor functions. On the other hand, the Tum 5-p53 fusion protein can increase the antitumor effect by consuming part of the MMP. To test this hypothesis, we constructed the hypoxia expression vector pET28-Pvhb-pelB-SUMO-Tum 5-MMP-p18-p53 and transferred the vector into the tumor-targeting bacterium EcN to obtain the engineered bacterium EcN (Tum 5-p53). The in vivo antitumor efficacy of the engineered strain was then studied. Although there is no direct evidence showing that the Tum 5-p53 fusion protein is successfully cleaved by MMPs in the tumor region, the excellent antitumor activity of EcN (Tum 5-p53) indicated that the antitumor efficacy of Tum 5-p53 bifunctional protein does indeed perform significantly better than the p53 protein or Tum-5 protein alone.

In a previous study, B16 tumor-bearing mice were injected intraperitoneally with 5 × 10^6^ CFUs/100 μL of EcN (Lux), and the tumor-targeting ability of EcN was detected using an in vivo imaging system [13]. The results indicated that the bioluminescence signal can be detected in the tumor area on the third day after bacterial injection. In this study, the SMMC-7721 tumor-bearing nude mice were intravenously injected with 5 × 10^6^ CFUs/100 μL of EcN (Lux), and a significant bioluminescence signal was also detected in the solid tumor area on the third day, which is delayed with the minimum time required for the documented EcN colonization of the tumor area. Dino et al. found that significant bioluminescence signals were detected in the tumor areas by in vivo imaging systems within 48 h after intravenous injection of Symbioflor-2 into murine CT26 colon cancer cell tumor-bearing mice [38]. Zhang et al. found that EcN can accumulate in the tumor areas of murine 4 T1 breast cancer tumor-bearing mice within 1–7 days after EcN (GFP) intravenous injection [14].

In the present study, although no signal was detected by the in vivo imaging system 24 h after tumor injection in the SMMC-7721 tumor-bearing nude mice, this does not indicate that there was no bacterial aggregation in the tumor area at that time. We speculate that the reason for this phenomenon is that the reporter gene *Lux*CDABE is a Lux operon cloned directly from the genome of *Photorhabdus luminescens*. As no strong promoter replacement was performed on this operon, the minimum bacterium concentration that caused EcN (Lux) to be detected by the bioimaging system was 2 × 10^6^ CFUs. Given that the operon was not replaced by a strong promoter, the bacterial concentration of EcN (Lux) that could be detected by the living body imaging system was high.

In vivo antitumor activity of the three engineered strains EcN (Tum-5), EcN (p53), and EcN (Tum 5-p53) on SMMC-7721 cells was also evaluated using tumor volume and tumor weight. Animal experiment results showed that there was no significant change in tumor volume and weight in nude mice in the PBS and EcN groups, indicating that wild-type EcN had no significant inhibitory effect on SMMC-7721 tumors. The tumor volume of EcN (Tum-5)-, EcN (p53)-, and EcN (Tum 5-p53)-treated mice decreased to varying degrees; the tumor weight was significantly reduced; and the Tum 5-p53 bifunctional protein exhibited significant antitumor efficacy. Compared with the PBS group, the tumor inhibition rate of the EcN (Tum 5-p53) group was as high as 69.47% (*P* < 0.05) in tumor volume and as high as 62.5% (*P* < 0.05) in tumor weight. The weight changes of mice in the PBS, EcN, EcN (Tum-5), EcN (p53), and EcN (Tum 5-p53) groups tended to stabilize during the entire course of treatment. There was no significant difference in pathological morphology of the main immune organs. These results indicated that EcN probiotics exhibit a safety profile upon systemic infection.

## Conclusions

In summary, the in vivo and in vitro antitumor effects of the Tum 5-p53 bifunctional proteins were examined. The tumor-targeting characteristics of EcN were investigated using luciferase *Lux*CDABE operon. Tum 5-p53 bifunctional proteins were initially constructed and then delivered to solid tumor regions by using the targeted transporter EcN for cancer therapy. Our findings provided a foundation for tumor-targeted therapy and demonstrated that the gene delivery of Tum 5-p53 bifunctional proteins to solid tumors could be an effective strategy for cancer therapy.

## Materials and methods

### Animal and cell culture

All tumor cells were cultured at 37 °C and in 5% CO_2_ atmosphere in RPMI 1640 supplemented with 10% heat-inactivated fetal bovine serum containing 100 U/mL penicillin and 100 μg/mL streptomycin. Male BALB/c nude mice aged 4–5 weeks and weighing 18–20 g were purchased from Hunan Slack Jingda Animal Experimental Company with license number of SYKX (Hunan) 2014–0006. BALB/c nude mice were fed under specific pathogen-free conditions for at least 7 days to adapt to the new environment. All animal experiments followed the National Institutes of Health Guide for the Care and Use of Laboratory Animals and were approved by the Animal Ethics Committee of Hunan Normal University.

### Gene cloning and soluble expression of p53

The mRNA of human breast cancer cell line MCF-7 was extracted, and the cDNA was prepared according to the manual accompanying the ABI reverse transcription kit. DNA sequences of *p53* were obtained by PCR technology. The heterologous p53 protein needs to penetrate the cell membrane to enter the cytoplasm and migrate to the cell nucleus to exert its antitumor activity. Taylor et al. proved that the amino acid 50–67 (p18) of azurin is the protein transduction domain responsible for transmembrane ability. The fusion expression of p53 and p18 peptide confers the ability of p53 protein to cross the cell membrane into the cytoplasm and allow the p53 protein to be solubly expressed in the prokaryotic expression system. The p18 double strands were ligated with pET28a vector at 16 °C overnight after digestion with *Nco* I + *Hind* III and transformed into *E. coli* GB2005. The transformants were selected and identified by enzyme digestion to construct the pET28a-p18 vector. The *p53* gene products were ligated with pET28a-p18 vector at 16 °C overnight after digestion with *Hind* III + *Xho* I and transformed into *E. coli* GB2005 to construct the p53-inducible expression vector pET28a-p18-p53. The correctly sequenced recombinant plasmid pET28a-p18-p53 was transformed into *E. coli* BL21 (DE3) to obtain the induced expression strain *E. coli* BL21(DE3)/pET28a-p18-p53. Soluble analysis of p53 protein was performed after induction with 0.3 mM IPTG. The supernatants and pellets were analyzed by sodium dodecyl sulfate–polyacrylamide gel electrophoresis (SDS-PAGE). The recombinant protein was cut with a blade and identified by LTQ-XL mass spectrometry (Thermo Fisher) after proteolysis.

### Construction of bifunctional tum 5-p53 protein

Based on previous studies, the primers were designed to construct different Tum-5 and p53 fusion expression vectors. The p53-F-*Hind* III and p53-PLGLWA-R primers were used to amplify the *p53* fragment with pET28a-p18-p53 plasmid DNA as a template. PLGLWA-Tum 5-F and Tum 5-R-*Xho* I were used to amplify the *Tum*-*5* fragment with pET28a-Tum 5 plasmid DNA as a template. Finally, the *p53*-*MMP*-*Tum 5* fragment was amplified by overlapping PCR with p53-F-*Hind* III and Tum 5-R-*Xho* I primers using *p53* and *Tum-5* fragments as the templates. The *p53-MMP-Tum 5* fragment was inserted into pET28a-p18 after digestion with *Hind* III + *Xho* I. The pET22b-p18-p53-MMP-Tum 5, pSmartI-p18-p53-MMP-Tum 5, and pSmartI-Tum 5-MMP-p18-p53 plasmids were constructed using the same method. Four sequenced recombinant plasmids were transformed into *E. coli* BL21 (DE3) to obtain the induced expression strains. The supernatant protein and pellet protein of induced expression strains were analyzed by SDS-PAGE after IPTG induction.

### Cytotoxicity of recombinant proteins against cancer cells

When cells occupied 70–80% of the bottom of the dish, they were digested with 500 μL of trypsin at 37 °C, and 1 mL of cell culture medium per dish was added to stop digestion. The cells were dissipated into single-cell suspensions and collected after centrifugation at 1000 rpm for 5 min. The cells were resuspended in the medium and adjusted to 8000 cells/100 μL in the 96-well plate. Recombinant proteins of different concentration gradients were added to each well after the cells were completely adherent, and each concentration had 8–10 replicates. After 48 or 72 h, CCK-8 solution was carefully added to each well and incubated for 2 h. The absorbance of each well at 450 nm was measured with a microplate reader to calculate the inhibition effect of the recombinant protein on the tumor cells.

### Construction of tumor-targeting bacteria EcN expression strains

The *Vitreoscilla* hemoglobin gene promoter *Pvhb* was amplified from pET-28a-Pvhb-pelB-asp (Lab store). Meanwhile, the *p18*-*p53* fragment was amplified from pET28a-p18-p53, and *Tum 5-MMP-p53* was amplified from pSmartI-Tum 5-MMP-p18-p53 (constructed in this study). *Pvhb-pelB-p18-p53* and *Pvhb-pelB-Tum 5-MMP-p18-p53* fragments were obtained by overlapping extension PCR and inserted into pET28a after digestion with *Apa* I + *Xho* I. The sequenced vectors were transformed into EcN by electroporation and named EcN (p53) and EcN (Tum 5-p53). EcN, EcN (28a), EcN (p53), and EcN (Tum 5-p53) were cultured in LB medium for 10 h. Then, SDS-PAGE and Western blot analysis were used to confirm the expression of proteins of interest by using Anti-6 × His rabbit polyclonal antibody.

### In vivo distribution of EcN and antitumor effects of the engineered bacteria

For the xenograft tumor model, 1 × 10^7^ SMMC-7721 cancer cells suspended in 100 μL of PBS were injected subcutaneously into the right axillary region of the BALB/c nude mice. To monitor bacteria distribution in mice after injection, a vector for the constitutive expression of *lux* was constructed by inserting the *lux*CDABE operon from *Photorhabdus luminescens* into the pET-28a vector. Then, the pET-28a-Lux plasmid was transformed into EcN by electroporation. After the tumor grew to a suitable size, the tumor-bearing nude mice and normal nude mice were intravenously injected with 5 × 10^6^ CFUs/100 μL EcN (Lux). Subsequently, the distribution of EcN (Lux) in the animal body was detected using an in vivo imaging system (IVIS, Calipers). The animals were sacrificed 72 h after injection. The tumors, liver, kidney, spleen, lung, heart, intestine, and skin of normal nude mice and tumor-bearing nude mice were dissected to detect the distribution of bacteria in these organs. The tumor-bearing nude mice were randomly divided into five groups (six mice per group). After overnight cultivation of EcN, EcN (Tum-5), EcN (p53), and EcN (Tum 5-p53), the bacteria were transferred to LB liquid media at a 2% v/v inoculum and cultivated at 37 °C for 5 h before collection. The cells were washed with sterile PBS for three times and diluted to a concentration of 5 × 10^6^ CFUs/100 μL. The mice were intravenously injected with sterilized PBS, EcN, EcN (p53), EcN (Tum-5), or EcN (Tum 5-p53) every week. During the experiment, each group of mice were weighed every 3 days, and the longest diameter and vertical maximum diameter of the mouse tumor were recorded every 3 days using vernier calipers. After the end of the experiment, mice were dissected to obtain the tumor, liver, kidney, spleen, lung, and heart. Tumor volume (TV) was calculated according to the formula: TV (mm^3^) = d^2^ x D/2, where d and D are the shortest and the longest diameter, respectively. The tumors of each group were fixed with 4% paraformaldehyde for H&E staining, immunohistochemistry (IHC), and immunofluorescence (IF). The liver, kidney, spleen, lung, and heart were fixed with 4% paraformaldehyde for H&E staining.

### Statistical analysis

SPSS statistics version 21.0 was used for all statistical analyses. Significant differences for continuous variables of non-normal distribution were determined using the Wilcoxon rank sum test. A probability value of *P* < 0.05 was considered statistically significant.

## Additional file

Additional file 1: **Table S1.** Primers used in PCR amplification. **Figure S1.** Agarose gel electrophoresis of human *p53* gene amplification products. **Figure S2.** Construction and identification of recombinant plasmid pET28a-p18. **Figure S3.** Construction and identification of plasmid pET28a-p18-p53. **Figure S4.** Purification and identification of recombinant p53 protein. **Figure S5.** Inhibitory effect of recombinant p53 protein on different tumor cells. **Figure S6.** The construction program of hypoxia expression vector pET28a-Pvhb-pelB-p18-p53. **Figure S7.** Construction of p53 hypoxia expression vector pET28a-Pvhb-pelB-p18-p53. **Figure S8.** Construction of *p53* and *Tum-5* fusion expression vector. **Figure S9.** Construction of *Tum-5* and *p53* fusion expression vector. **Figure S10.** Morphological changes of different tumor cells after recombinant Tum 5-p53 treated (100×). **Figure S11.** The construction process diagram of hypoxia expression vector pET28a-Pvhb-pelB-SUMO-Tum 5-MMP-p53. **Figure S12.** Construction of recombinant plasmid pET28a-Pvhb-pelB-SUMO-Tum 5-MMP-p53 (DOC 1943 kb)

## Data Availability

All data generated or analysed during this study are included in this published article and its supplementary information files.

## Abbreviations

CFU: Colony-forming unit
EcN: *Escherichia coli* Nissle 1917
H&E: Hematoxylin and eosin staining
IF: Immunofluorescent
IHC: Immunohistochemistry
IPTG: Isopropyl-β-d-thiogalactoside
Ki-67: Nuclcar associated antigen Ki- 67
MMP: Matrix metalloproteinase
PBS: Phosphate buffer solution
SDS–PAGE: Sodium dodecyl sulfate–polyacrylamide gel electrophoresis
SPF: Specific pathogen free
SUMO: Small ubiquitin-related modifier
