# Supplementary material for: Intestinal probiotics E. coli Nissle 1917 as a targeted vehicle for delivery of p53 and Tum-5 to solid tumors for cancer therapy
Source: J Biol Eng. 2019 Jun 28;13:58. doi: 10.1186/s13036-019-0189-9 (PMC6599283; doi:10.1186/s13036-019-0189-9)
Supplement: Supplementary file 1 — Table S1. Primers used in PCR amplification. Figure S1. Agarose gel electrophoresis of human p53 gene amplification products. Figure S2. Construction and identification of recombinant plasmid pET28a-p18. Figure S3. Construction and identification of plasmid pET28a-p18-p53. Figure S4. Purification and identification of recombinant p53 protein. Figure S5. Inhibitory effect of recombinant p53 protein on different tumor cells. Figure S6. The construction program of hypoxia expression vector pET28a-Pvhb-pelB-p18-p53. Figure S7. Construction of p53 hypoxia expression vector pET28a-Pvhb-pelB-p18-p53. Figure S8. Construction of p53 and Tum-5 fusion expression vector. Figure S9. Construction of Tum-5 and p53 fusion expression vector. Figure S10. Morphological changes of different tumor cells after recombinant Tum 5-p53 treated (100×). Figure S11. The construction process diagram of hypoxia expression vector pET28a-Pvhb-pelB-SUMO-Tum 5-MMP-p53. Figure S12. Construction of recombinant plasmid pET28a-Pvhb-pelB-SUMO-Tum 5-MMP-p53 (DOC 1943 kb) [file 13036_2019_189_MOESM1_ESM.doc]

**Supporting Information**

**Intestinal probiotics** ***E.coli* Nissle 1917 as a targeted vehicle for delivery of p53 and Tum-5 to solid tumors for cancer therapy**

**Content:**

1. **Primers**
2. **Supporting figures**
3. **Primers**

**Table 1. Primers used in PCR amplification**

（Underline: restriction site）

| **Primers** | **DNA Sequence(5′→ 3′)** |
| --- | --- |
| *Nco* I-p18-F  p18-*Hind* III-R | CATGGTA CTGAGCACCGCCGCCGACATGCAGGGCGTGGTCA  CCGACGGCATGGCTTCCGGC A  AGCTT GCCGGAAGCCATGCCGTCGGTGACCACGCCCTGCAT  GTCGGCGGCGGTGCTCAG TAC |
| p53-F-*Hind* III  p53-R-*Xho* I | CCC AAGCTT ATGGA GGAGCCGCAGTCAGA  CCG CTCGAG GTCTG AGTCAGGCCCTTCTG |
| Pvhb-pelB-p18-F  p53-His-R | TGCCTACGGCAGCCGCTGGATTGTTATTACTCGCTGCCCAACCAGCGATGGCT CtgagcaccgccgccgacaT  TTTCGGGCTTTGTTAGCAGCCGGATCTCAGTGGTGGTGGTGGTGGTGCTCGAG GTCTGAGTCAGGCCCTTCTG |
| p53-PLGLWA-R  PLGLWA-p18-p53 | TGCCCATAATCCTAATGGGTCTGAGTCAGGCCCTTCTG  CCATTAGGATTATGGGCACTGAGCACCGCCGCCGACAT |
| PLGLWA-Tum 5-F  Tum 5-R-*Xho* I | CCATTAGGATTATGGGCAGGGttttcttttCtttttgt  CCGCTCGAGGGCGATCGCAGGACCTTCAC |
| *Bam*H I-p18-F | CGCGGATCCCTGAGCACCGCCGCCGACAT |
| Tum 5-F-*Bam*H I  Tum 5-PLGLWA-R | CGCGGATCCGGGTTTTCTTTTCTTTTTGT  TGCCCA TAA TCCTAATGGGGCGATCGCAGGACCTTCAC |
| VHB-F-*Apa* I  VHB-R-SUMO  SUMO-F | TGGGGGCCCTAGCTTACAGGACGCTGGGG  GAGTCCGACATATGGTGATGGTGATGGTGACCCATAGCCATCGCTGGTTGGGCAG  ATGGGTCACCATCACCATCACC |

1. **Supporting figures**


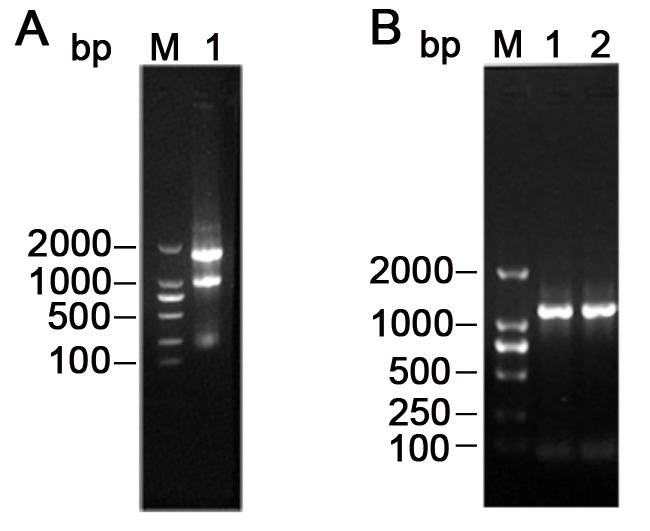


**Fig. S1** Agarose gel electrophoresis of human *p53* gene amplification products

(A) Electrophoresis analysis of total RNA from human breast cancer cell MCF-7. M: DL2000 DNA Marker; lane 1: Total RNA of MCF-7. (B) Electrophoresis analysis of human *p53* gene amplification products. M: DL2000 DNA Marker; lane 1-2: Amplification products of *p53*.


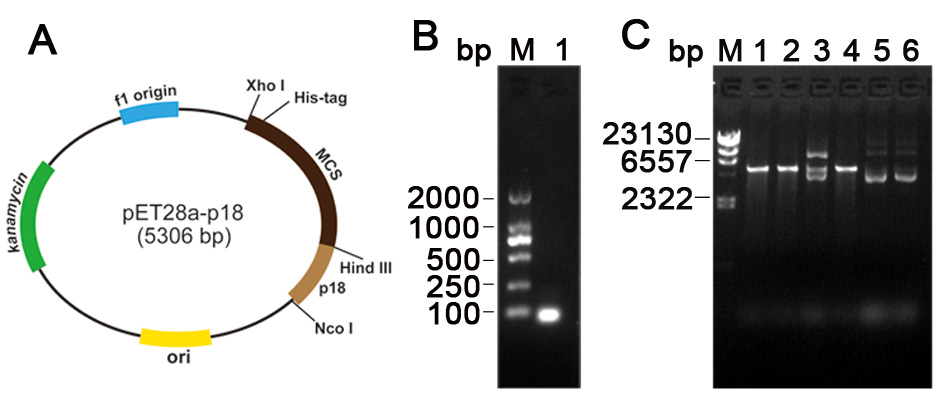


**Fig. S2** Construction and identification of recombinant plasmid pET28a-p18

(A) Vector map of pET28a-p18. (B) Electrophoresis analysis of double-strand p18. M: DL2000 DNA Marker; lane 1: double-strand p18. (C) Restriction enzyme digestion analysis of pET28a-p18 plasmid. M: λ*-Hind* III DNA Marker; lane 1 and lane 4: pET28a, pET28a-p18 were digested with *Xho* I; lane 2 and lane 5: pET28a, pET28a-p18 were digested with *Bam*H I; lane 3: pET28a plasmid; lane 6: pET28a-p18 plasmid.


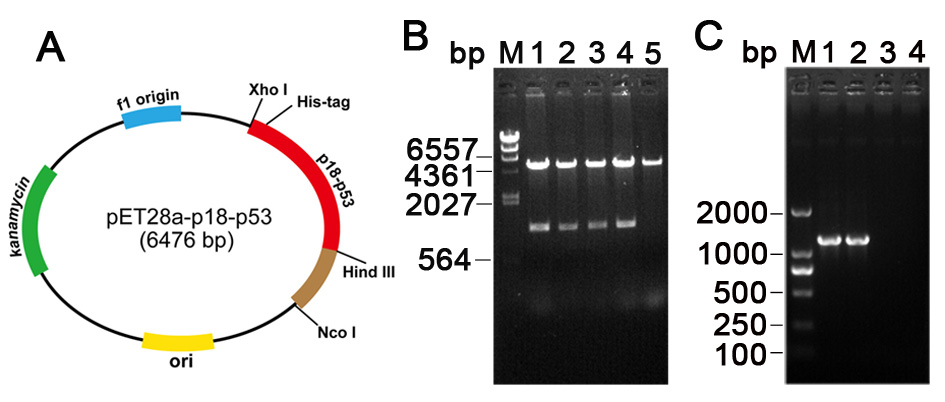


**Fig. S3** Construction and identification of plasmid pET28a-p18-p53

(A) Vector map of pET28a-p18-p53. (B) Restriction enzyme analysis of pET28a-p18-p53 in *E.coli* GB2005. M: λ*-Hind* III DNA Marker; lane 1-4: Restricted DNA products after *Hind* III + *Xho* I digestion; lane 5: Digested products of pET28a-p18. (C) PCR identification of p53 in *E.coli* GB2005. M: DL2000 DNA Marker; lane 1-2: PCR products of pET28a-p18-p53 plasmid; 3-4: PCR products of pET28a-p18 plasmid.


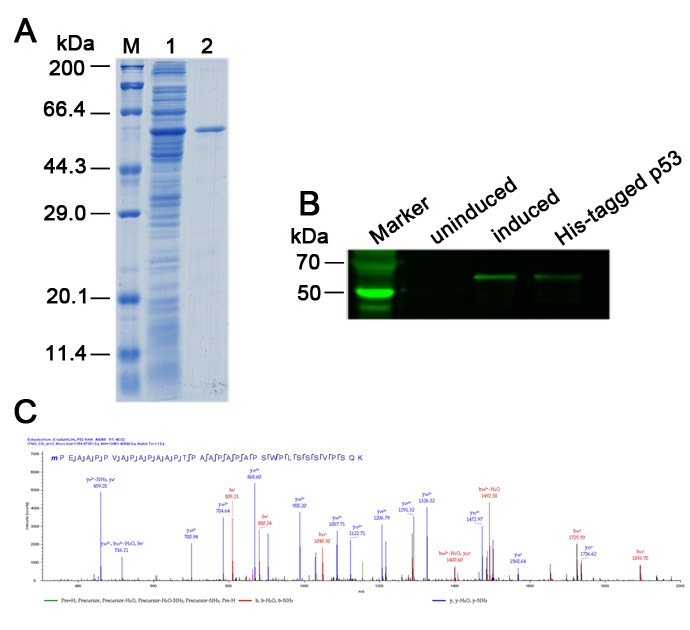


**Fig. S4** Purification and identification of recombinant p53 protein

(A) Desalting products of p53 were detected by SDS–PAGE. M: protein molecular weight standard; 1: cell lysate of *E.coli* BL21 (DE3)/pET28a-p18-p53 after IPTG induction; 2: desalination product of p53. (B) Western blot analysis of p53 expression in *E.coli* BL21 (DE3). (C) LC-MS analysis of recombinant p53 protein.


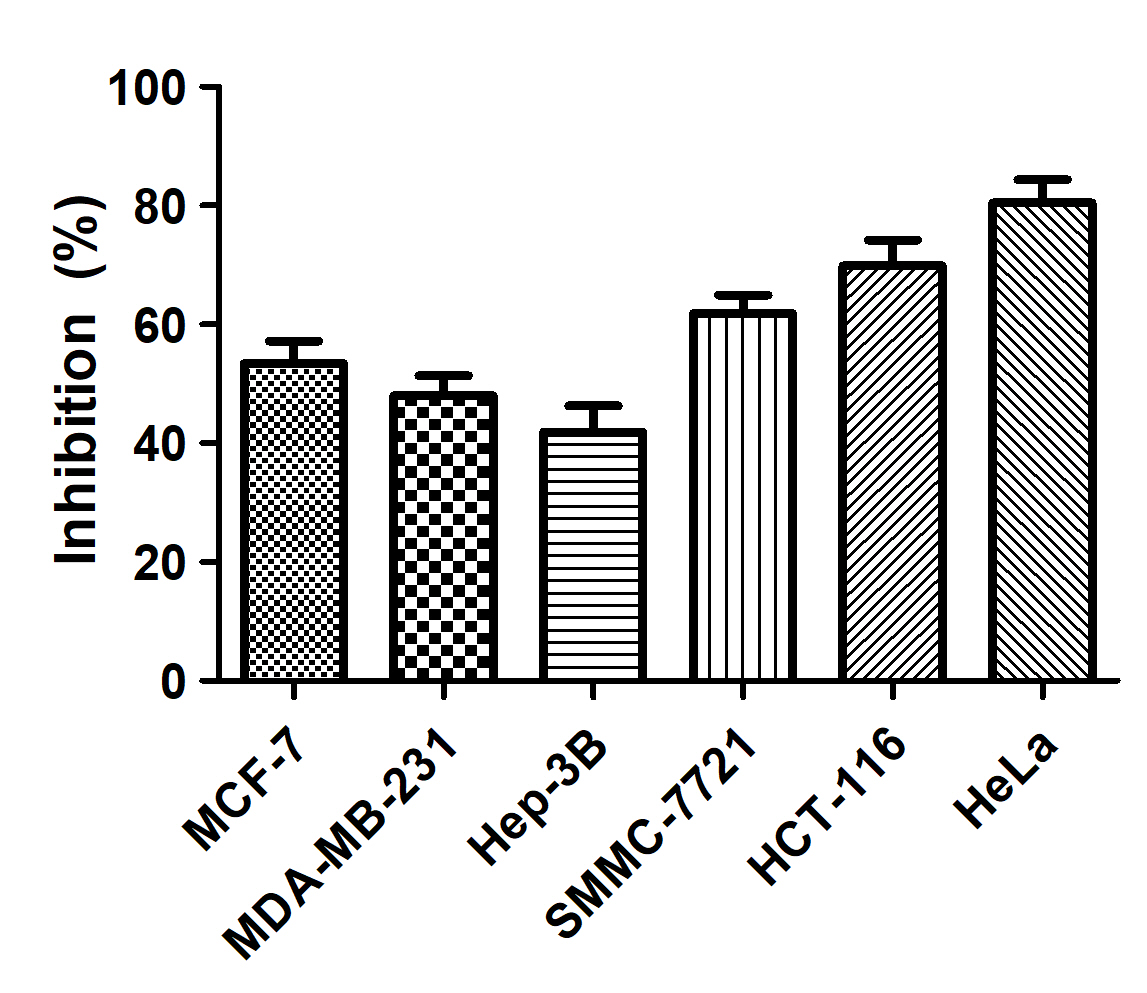


**Fig. S5** Inhibitory effect of recombinant p53 protein on different tumor cells


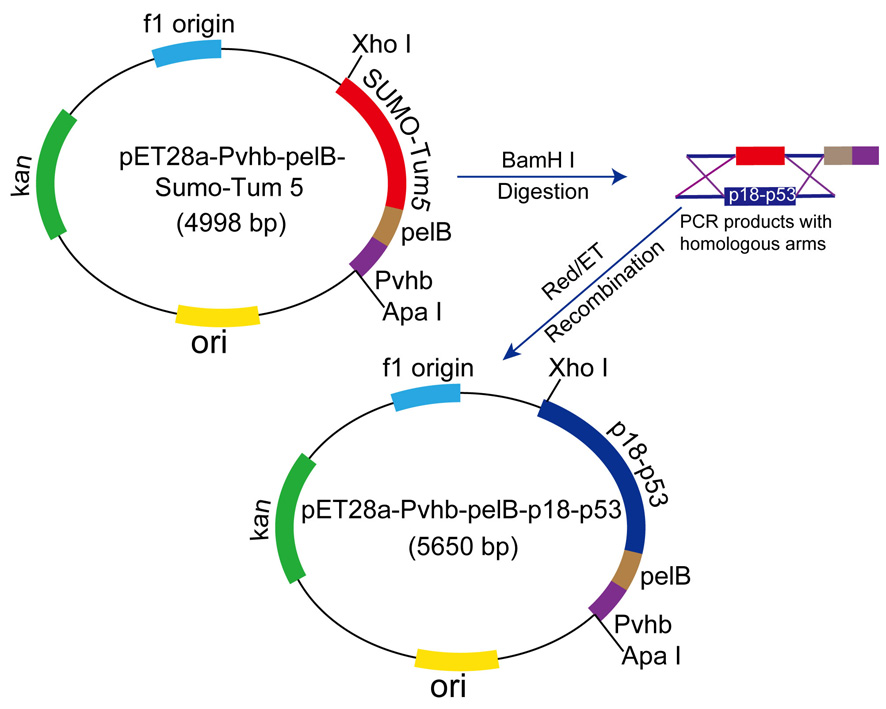


**Fig. S6** The construction program of hypoxia expression vector pET28a-Pvhb-pelB-p18-p53


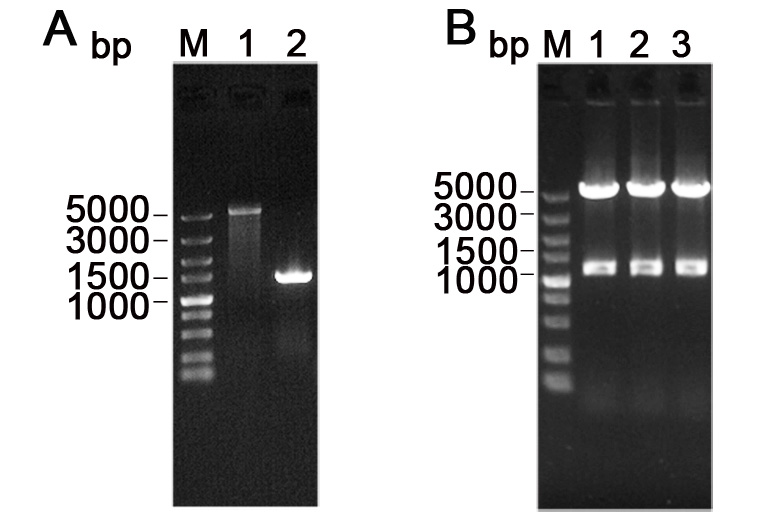


**Fig. S7** Construction of p53 hypoxia expression vector pET28a-Pvhb-pelB-p18-p53

(A) PCR amplification of p18-p53 with homology arm and restriction enzyme digestion of the vector. M: DL5000 DNA Marker; lane 1: pET28a-Pvhb-pelB-SUMO-Tum 5 plasmid was digested with *Bam*H I; lane 2: PCR products of p18-p53 with homology arm. (B) Restriction map of recombinant plasmid pET28a-Pvhb-pelB-p18-p53. M: DL5000 DNA Marker; lane 1-3: pET28a-Pvhb-pelB-p18-p53 plasmid was digested with *Hind* III + *Xho* I.

**
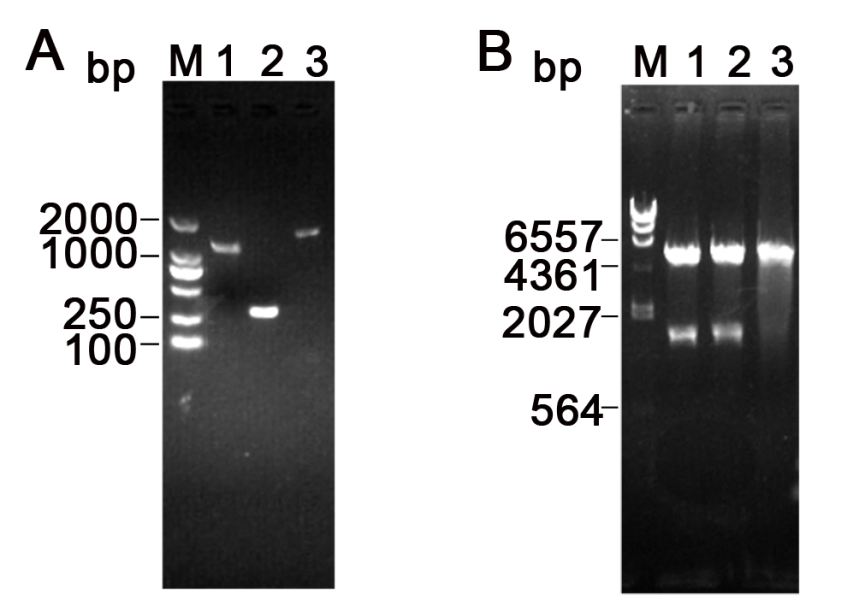
**

**Fig. S8 Construction of *p53* and *Tum-5* fusion expression vector**

(A) Electrophoresis analysis of p53-MMP-Tum 5 fragment. M: DL2000 DNA Marker; lane 1: Amplification products of p53-PLGLWA fragment; lane 2: Electrophoresis analysis of PLGLWA-Tum 5 fragment; lane 3: p53-MMP-Tum 5 fragment was obtained by fusion PCR. (B) Restriction analysis of pET28a-p18-p53-MMP-Tum 5 and pET22b-p18-p53-MMP-Tum 5 plasmids. M: λ*-Hind* III DNA Marker; lane1: pET28a-p18-p53-MMP-Tum 5 was digested with *Hind* III + *Xho* I; lane 2: pET22b-p18-p53-MMP-Tum 5 was digested with *Hind* III + *Xho* I；lane 3: Digestion products of empty vector.


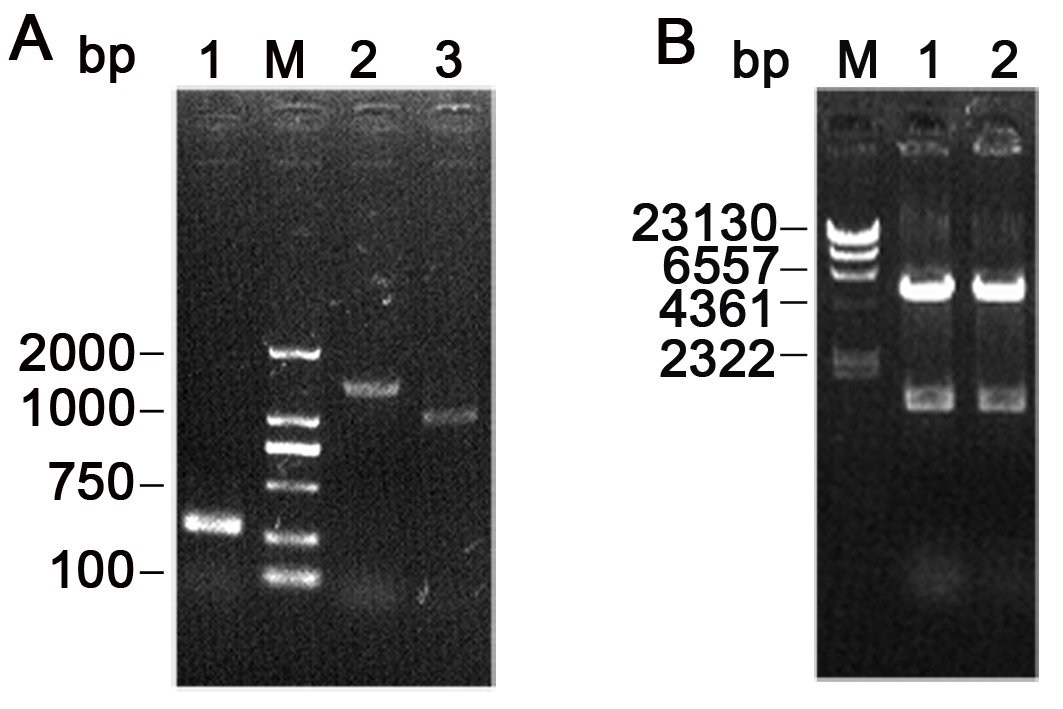


**Fig. S9** Construction of *Tum-5* and *p53* fusion expression vector

(A) Electrophoresis analysis of Tum 5-MMP-p53 fragment amplification products. M: DL2000 DNA Marker; lane 1: Electrophoresis analysis of Tum 5-PLGLWA fragment; lane 2: Tum 5-MMP-p53 fragment was obtained by fusion PCR; lane 3: Amplified products of PLGLWA-p53 fragment. (B) Restriction analysis of pSmartI-p18-p53-MMP-Tum 5 and pSmatI-Tum 5-MMP-p18-p53 plasmids. Lane 1-2: pSmartI-p18-p53-MMP-Tum 5 (lane 1) and pSmatI-Tum 5-MMP-p18-p53 (lane 2) were digested with *Bam*H I + *Xho* I.


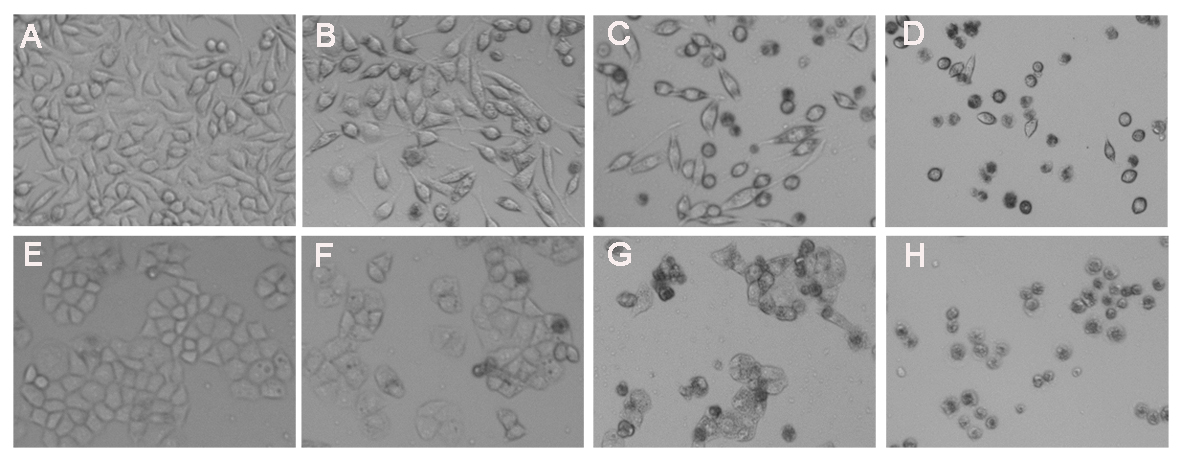


**Fig. S10 Morphological changes of different tumor cells after recombinant Tum 5-p53 treated (100×)**

Human hepatoma cells SMMC-7721 treated with PBS (A) and Tum 5-p53 for 20 µg/mL (B), 40 µg/mL (C), 80 µg/mL (D); Human [cervical](../../../../C:/Users/lenovo-06/AppData/Local/youdao/Dict/Application/7.5.2.0/resultui/dict/%3Fkeyword=cervical)[carcinoma](../../../../C:/Users/lenovo-06/AppData/Local/youdao/Dict/Application/7.5.2.0/resultui/dict/%3Fkeyword=carcinoma) cells treated with PBS (E) and Tum 5-p53 for 20 µg/mL (F), 40 µg/mL (G), 80 µg/mL (H).


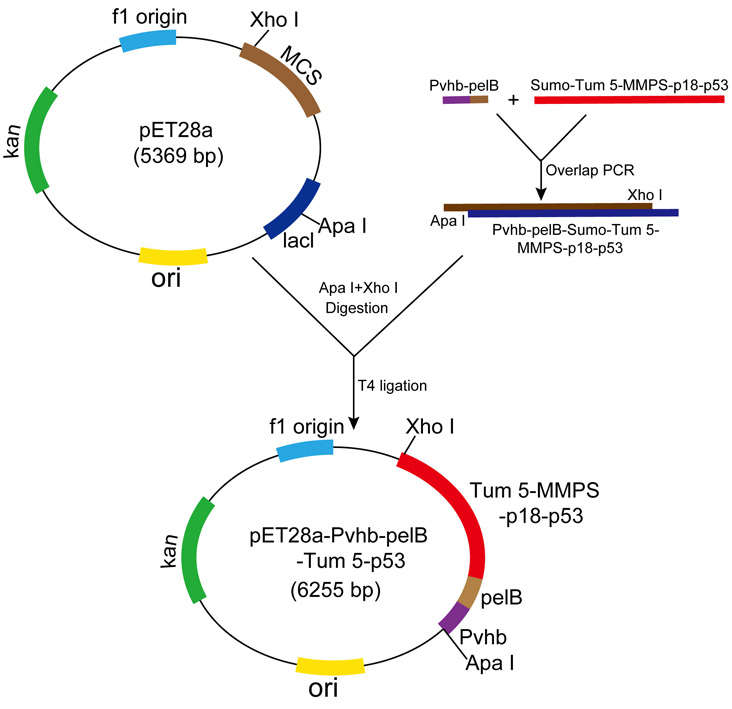


**Fig. S11** The construction process diagram of hypoxia expression vector pET28a-Pvhb-

pelB-SUMO-Tum 5-MMP-p53


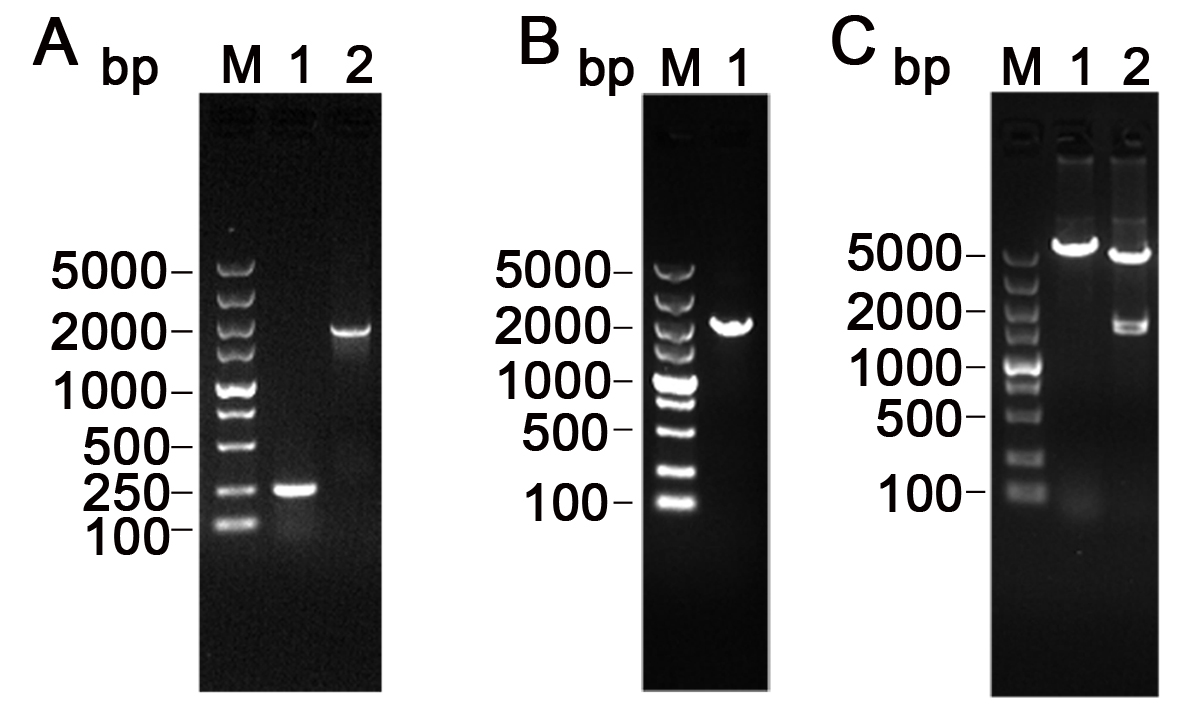


**Fig. S12** Construction of recombinant plasmid pET28a-Pvhb-pelB-SUMO-Tum 5-MMP-p53

(A) PCR amplification of Pvhb-pelB and Tum 5-MMP-p53 fragments. M: DL5000 DNA Marker; lane 1: PCR products of Pvhb-pelB; lane 2: PCR products of Tum 5-MMP-p53. (B) PCR amplification of Pvhb-pelB-SUMO-Tum 5-MMP-p53 fragment. M: DL5000 DNA Marker; lane 1: Pvhb-pelB-SUMO-Tum 5-MMP-p53 fragment was obtained by fusion PCR. (C) PCR identification of pET28a-Pvhb-pelB-SUMO-Tum 5-MMP-p53. lane 1: pET28a plasmid was digested with *Bam*H I + *Xho* I; lane 2: pET28a-Pvhb-pelB-SUMO-Tum 5-MMP-p53 plasmid was digested with *Bam*H I + *Xho* I.
